# The Role of Novel Autoantibodies in the Diagnostic Approach and Prognosis of Patients with Raynaud’s Phenomenon

**DOI:** 10.31138/mjr.31.4.427

**Published:** 2020-12-28

**Authors:** Vasiliki Koulouri, Adrianos Nezos, Nikolaos Marketos, Evangelia Argyriou, Kyriaki Boki, Dimitrios Ioakimidis, Michalis Koutsilieris, Clio P. Mavragani

**Affiliations:** 1 Department of Physiology, School of Medicine, National and Kapodistrian University of Athens, Athens, Greece; 2 Rheumatology Outpatient Department, Henry Dunant Hospital Centre, Athens, Greece; 3 Rheumatology Unit, Sismanogleio General Hospital, Athens, Greece; 4 Rheumatology Outpatient Unit, Department of Pathophysiology, School of Medicine, National and Kapodistrian University of Athens, Laikon Hospital, Athens, Greece

**Keywords:** Autoantibodies, Raynaud’s phenomenon, systemic autoimmune rheumatic disease

## Abstract

Raynaud’s phenomenon (RP) is a condition characterised by distinct colour changes of the digits upon exposure to sympathomimetic conditions, such as cold temperature. It can be either primary or secondary, depending on whether it presents alone or as part of an underlying disorder. One of the most common causes of secondary RP are systemic autoimmune rheumatic diseases (SARDs), in which RP may precede the onset of other autoimmune features by many years. Thus, timely and accurate recognition of secondary RP is of great importance as it alters patient management and prognosis. An important step in the diagnostic approach of RP is the detection of antinuclear antibodies (ANAs) by indirect immunofluorescence. However, identification of specific autoantibodies is not yet common practice, though many of them have shown important clinical associations. Moreover, the role of some autoantibodies has not yet been elucidated, given their relatively recent discovery and low reported prevalence rates in autoimmune population. The goal of this study is to reveal clinical associations of these novel autoantibodies in SARDs through the application of an extended serology workup in patients presenting with RP.

## INTRODUCTION

Raynaud’s phenomenon (RP) is a term encompassed to describe a distinct colour change (white, blue, red) of the digits, as a result of irregular vasoconstriction in response to exposure to cold temperatures. It is considered to be either primary or secondary in the absence or presence of an underlying disorder respectively.^1^ Secondary RP has been associated with several systemic autoimmune rheumatic diseases (SARDs), mainly systemic sclerosis (SSc), systemic lupus erythematosus (SLE), Sjögren’s syndrome (SS), mixed and undifferentiated connective tissue disorder (MCTD and UCTD) and idiopathic inflammatory myopathies (IIM).^2–8^

Recognition of secondary RP is of great importance, as the phenomenon might precede the diagnosis of a well-established SARD by many years, as is the case in SSc.^2^ According to a comprehensive meta-analysis, approximately 12.6% of patients diagnosed with primary RP eventually “transitioned” to secondary RP, mostly attributed to an underlying SARD.^9^ Thus, early detection of secondary Raynaud’s would allow timely diagnosis of SARD and establishment of appropriate diagnostic approach, monitoring, and eventually of tailored therapeutic modalities, ultimately contributing to better outcomes.

An imperative step in the diagnostic approach of RP is the detection of antinuclear antibodies (ANAs). Aside from their utility in diagnosis, several autoantibodies have been shown to be associated with disease activity and complications, specific organ damage, response to treatment and even underlying malignancy.^10^ In the case of RP, most of the RP-associated autoantibodies are SSc associated autoantibodies (SAA), as a result of the universal presence of the phenomenon in this disease.^2^ Specifically, ANAs and some SAAs (anti-Scl-70, anti-Th/To, anti-RNA polymerase, anti-centromere) have been shown to be independent prognostic factors of both SSc development in RP patients and abnormal capillaroscopic patterns.^11,12^ Positive ANAs and presence of anti-Scl-70 have been associated with poor prognosis in female patients with RP,^13^ while in children presenting with RP, positive ANAs are a significant prognostic factor of future SARD development.^14^ On the other hand, absence of both ANA and pathologic capillaroscopy almost excludes the possibility of progress to SSc.^11^

Antibodies targeting tRNA synthetases (anti-ARS) are indirectly linked with RP as part of the antisynthetase syndrome (AS), an autoimmune disease characterised by the presence of myositis, interstitial lung disease, mechanic’s hands, fever, arthritis, and RP, to varying degrees.^15,16^ A patient with RP and anti-ARS positivity meets Connor’s criteria for AS^15^ and thus calls for a more thorough look into potential muscle and/or lung involvement. Specifically, anti-PL-7 and anti-PL-12 have been more commonly associated with early respiratory insult rather than myositis.^17^ Interestingly, myositis-associated autoantibodies (MAAs) have shown stronger connection to RP in patients with myositis compared to myositis specific antibodies (MSAs),^18^ probably due to the fact that MAAs are also clinically associated with SSc. Presence of anti-U1 RNP is a sine qua non of MCTD diagnosis^19^ and its association with RP is further confirmed in other SARDs such as SLE, SSc and UCTD.^3,20,21^ Both anti-U1 RNP and RP have been linked with respiratory involvement in SLE patients.^22^

Though detection of a wide array of autoantibodies has not yet been included in clinical practice, it seems to be an important tool in both diagnosis and overall patient management. For instance, a patient with RP and SAA positivity does not currently meet criteria for SSc but could meet the criteria for very early disease onset SSc (VEDOSS),^23, 24^ This group of patients could potentially benefit from early immunosuppressive treatment which eventually delay or prevent internal organ damage.^25^

## AIM OF THE STUDY

Detection of specific autoantibodies in RP may not only guide diagnosis but also assist with patient management, as a result of established clinical associations of these autoantibodies. However, our knowledge regarding novel autoantibodies is currently limited, in view of their relatively recent discovery and low reported prevalence rates in autoimmune populations. Therefore, our goal is to reveal the incidence and potentially valuable clinical associations of novel autoantibodies in patients presenting with RP, through the use of an extended standardized serologic array.

## MATERIALS AND METHODS

For our study, serum from patients presenting with RP will be tested in the Department of Physiology, School of Medicine, National and Kapodistrian University of Athens for the presence of a wide array of autoantibodies, once the patients have agreed to participate in this study and have signed informed consent. Patient referrals from collaborating centres (ie, Rheumatology Outpatient Department of Henry Dunant Hospital Centre and Rheumatology Unit of Sismanogleio General Hospital) may also be included. Information regarding their medical history, clinical examination and basic laboratory values will be archived. The study will include subjects over 18 years of age.

Detection of autoantibodies will be performed using the following immunoblot assays including an extensive autoantigen profile, according to manufacturer’s instructions (EUROIMMUN AG):

a) EUROLINE ANA profile 3: nRNP (U1)/Sm, Sm, Ro(SS-A),Ro-52, (La) SS-B, Scl-70, PM/Scl-100, Jo-1, CENP B, PCNA, dsDNA, Nucleosomes, Histones, Rib.P και AMA M2
b) EUROLINE Autoimmune Inflammatory Myopathies 16 Ag: Mi-2α, Mi-2β, TIF-1γ, MDA5, NXP2, SAE1, Ku, PM/Scl-100, PM/Scl-75, Jo-1, SRP, PL-7, PL-12, EJ, OJ και Ro-52
c) EUROLINE Systemic Sclerosis (Nucleoli) Profile: Scl-70, CENP A, CENP B, RP11, RP155, Fibrillarin, NOR90, Th/To, PM/Scl-100, PM/Scl-75, Ku, PDGFR και Ro52

Based on the overall assessment, the subjects will be then classified as suffering from either primary or secondary RP (pRP or sRP) and further examination (such as cardiac triplex, pulmonary function tests, high resolution computed tomography, electromyography or oesophageal manometry) may be ordered, depending on relevant indications. In one year follow up clinical evaluation will be conducted in all patients, while basic laboratory workup and imaging studies (where previously applied) will be repeated in the sRP group in order to assess disease progression. Statistical analysis will be performed by the end of the re-evaluation process. Approximately 100 patients are estimated to be included in the study and the protocol is expected to be completed in 30 months’ time.

## ABBREVIATIONS

ANAs: Antinuclear antibodies
anti-ARS: Anti-tRNA synthetases
AS: Antisynthetase syndrome
IIM: Idiopathic inflammatory myopathies
MAAs: Myositis associated autoantibodies
MCTD: Mixed connective tissue disorder
MSAs: Myositis specific autoantibodies
RP: Raynaud’s phenomenon
SARDs: Systemic autoimmune rheumatic diseases
SLE: Systemic lupus erythematosus
SS: Sjögren’s syndrome
SSc: Systemic sclerosis
SAA: SSc associated autoantibodies
UCTD: Undifferentiated connective tissue disorder
